# Supplementary material for: UBE3A-mediated p18/LAMTOR1 ubiquitination and degradation regulate mTORC1 activity and synaptic plasticity
Source: eLife. 2018 Jul 18;7:e37993. doi: 10.7554/eLife.37993 (PMC6063731; doi:10.7554/eLife.37993)
Supplement: Transparent reporting form [file elife-37993-transrepform.docx]

***eLife’s* transparent reporting form**

We encourage authors to provide detailed information *within their submission* to facilitate the interpretation and replication of experiments. Authors can upload supporting documentation to indicate the use of appropriate reporting guidelines for health-related research (see [EQUATOR Network](http://www.equator-network.org/%20)), life science research (see the [BioSharing Information Resource](https://biosharing.org/)), or the [ARRIVE guidelines](http://www.plosbiology.org/article/info:doi/10.1371/journal.pbio.1000412) for reporting work involving animal research. Where applicable, authors should refer to any relevant reporting standards documents in this form.

If you have any questions, please consult our Journal Policies and/or contact us: [editorial@elifesciences.org](mailto:editorial@elifesciences.org).

**Sample-size estimation**

- You should state whether an appropriate sample size was computed when the study was being designed
- You should state the statistical method of sample size computation and any required assumptions
- If no explicit power analysis was used, you should describe how you decided what sample (replicate) size (number) to use

Please outline where this information can be found within the submission (e.g., sections or figure legends), or explain why this information doesn’t apply to your submission:

No statistical method was used to pre-determine sample size; however, the sample size per experiment was based on our previous successful experiments and publications. In the figure legends (Figure 1A,E, 2F,G, 3A,C,D,E, 4B,D,F,G, 5B,D, 6C,D-E, 7A-B,E, 8B,D,E, Figure 1-figure supplement 1B,E, Figure 3-figure supplement 1B, Figure 4-figure supplement 1D, 2D,E, Figure 5-figure supplement 1A, Figure 6-figure supplement 1A,E, 2, Figure 8-figure supplement 1A,C) the n-number used is mentioned.

**Replicates**

- You should report how often each experiment was performed
- You should include a definition of biological versus technical replication
- The data obtained should be provided and sufficient information should be provided to indicate the number of independent biological and/or technical replicates
- If you encountered any outliers, you should describe how these were handled
- Criteria for exclusion/inclusion of data should be clearly stated
- High-throughput sequence data should be uploaded before submission, with a private link for reviewers provided (these are available from both GEO and ArrayExpress)

Please outline where this information can be found within the submission (e.g., sections or figure legends), or explain why this information doesn’t apply to your submission:

Details on technical/biological replicates are presented in figure legends (culture dishes, slices, animals, technical/biological replicates) and in the methods section under “P2/S2 Fractionation, Lysosomal Fractionation, and Western Blot Analysis”, “Immunoprecipitation and Denaturing Immunoprecipitation”, “In Vitro Ubiquitin Assay”, “His-ubiquitin Pull-down Assay”, and “Image Analysis and Quantification”.

We did not encounter any outliers and thus we did not exclude any animals/cells because of that. Criteria for omitting animals (AAV in vivo injection) is mentioned in the results section 6 and in the methods section under “Immunofluorescence”.

**Statistical reporting**

- Statistical analysis methods should be described and justified
- Raw data should be presented in figures whenever informative to do so (typically when N per group is less than 10)
- For each experiment, you should identify the statistical tests used, exact values of N, definitions of center, methods of multiple test correction, and dispersion and precision measures (e.g., mean, median, SD, SEM, confidence intervals; and, for the major substantive results, a measure of effect size (e.g., Pearson's r, Cohen's d)
- Report exact p-values wherever possible alongside the summary statistics and 95% confidence intervals. These should be reported for all key questions and not only when the p-value is less than 0.05.

Please outline where this information can be found within the submission (e.g., sections or figure legends), or explain why this information doesn’t apply to your submission:

The statistical analysis methods are generally described in a specific section in the end of the Methods section. The specific statistical tests, exact values of N, methods of multiple test correction, and exact p-values are described in detail in each figure legend.

(For large datasets, or papers with a very large number of statistical tests, you may upload a single table file with tests, Ns, etc., with reference to sections in the manuscript.)

**Group allocation**

- Indicate how samples were allocated into experimental groups (in the case of clinical studies, please specify allocation to treatment method); if randomization was used, please also state if restricted randomization was applied
- Indicate if masking was used during group allocation, data collection and/or data analysis

Please outline where this information can be found within the submission (e.g., sections or figure legends), or explain why this information doesn’t apply to your submission:

The statements of randomization are presented in the methods section under “Immunofluorescence”, “Intrahippocampal AAV Injection”, and “Dendritic Spine Analysis”.

The statements of blinding are presented in the methods section under “Image Analysis and Quantification” and “Dendritic Spine Analysis”.

**Additional data files (“source data”)**

- We encourage you to upload relevant additional data files, such as numerical data that are represented as a graph in a figure, or as a summary table
- Where provided, these should be in the most useful format, and they can be uploaded as “Source data” files linked to a main figure or table
- Include model definition files including the full list of parameters used
- Include code used for data analysis (e.g., R, MatLab)
- Avoid stating that data files are “available upon request”

Please indicate the figures or tables for which source data files have been provided:

All numerical data that are represented as a graph in a figure are presented in source data files, accompanied by any supporting statistical analysis. The full Western blots are also provided.
